# Comparative single-cell analysis reveals heterogeneous immune landscapes in adenocarcinoma of the esophagogastric junction and gastric adenocarcinoma

**DOI:** 10.1038/s41419-023-06388-6

**Published:** 2024-01-05

**Authors:** Jierong Chen, Qunsheng Huang, Yi-Qi Li, Zhi Li, Jiabin Zheng, Weixian Hu, Yuesheng Yang, Deqing Wu, Jin-Xin Bei, Bing Gu, Junjiang Wang, Yong Li

**Affiliations:** 1 Department of Gastrointestinal Surgery, Department of General Surgery, Guangdong Provincial People’s Hospital (Guangdong Academy of Medical Sciences), Southern Medical University, Guangzhou, 510080 China; 2 Department of Laboratory Medicine, Guangdong Provincial People’s Hospital (Guangdong Academy of Medical Sciences), Southern Medical University, Guangzhou, 510080 China; 3 State Key Laboratory of Oncology in South China, Guangdong Provincial Clinical Research Center for Cancer, Sun Yat-sen University Cancer Center, Guangzhou, 510060 China; 4 Department of General Surgery, The Affiliated Cancer Hospital of Zhengzhou University and Henan Cancer Hospital, Zhengzhou, 450000 China

**Keywords:** Gastric cancer, Tumour heterogeneity

## Abstract

Adenocarcinoma of the esophagogastric junction (AEG) is a type of tumor that arises at the anatomical junction of the esophagus and stomach. Although AEG is commonly classified as a subtype of gastric adenocarcinoma (GAC), the tumor microenvironment (TME) of AEG remains poorly understood. To address this issue, we conducted single-cell RNA sequencing (scRNA-seq) on tumor and adjacent normal tissues from four AEG patients and performed integrated analysis with publicly available GAC single-cell datasets. Our study for the first time comprehensively deciphered the TME landscape of AEG, where heterogeneous AEG malignant cells were identified with diverse biological functions and intrinsic malignant nature. We also depicted transcriptional signatures and T cell receptor (TCR) repertoires for T cell subclusters, revealing enhanced exhaustion and reduced clone expansion along the developmental trajectory of tumor-infiltrating T cells within AEG. Notably, we observed prominent enrichment of tumorigenic cancer-associated fibroblasts (CAFs) in the AEG TME compared to GAC. These CAFs played a critical regulatory role in the intercellular communication network with other cell types in the AEG TME. Furthermore, we identified that the accumulation of CAFs in AEG might be induced by malignant cells through FGF-FGFR axes. Our findings provide a comprehensive depiction of the AEG TME, which underlies potential therapeutic targets for AEG patient treatment.

## Introduction

Adenocarcinoma of the esophagogastric junction (AEG) is a term referring to adenocarcinomas that occur within 5 cm above and below the anatomical boundary line between the esophagus and stomach. In recent years, the incidence of AEG has been continuously increasing worldwide [1, 2]. Although AEG is commonly classified together with gastric adenocarcinoma (GAC) in cancer registration and clinical trials of targeted therapy, it differs from esophageal adenocarcinoma and gastric cancer in terms of genomics, proteomics, metabolomics, clinical pathological features, and treatment outcomes [3–7]. The two-year follow-up data of a most recent clinical trial (CheckMate 649) showed that PD-1 immunotherapy significantly improved the prognosis of patients with GAC, but not for those with AEG [8]. Therefore, elucidating the tumor immune microenvironment of AEG is of great clinical value for the treatment selection and prognosis of patients.

The developments in single-cell RNA sequencing (scRNA-seq) technology has brought about an accurate and in-depth manner for profiling the intra- and inter-tumoral heterogeneity in various cancers [9]. By identifying key cell subtypes and critical intercellular interactions, the scRNA-seq studies revealed the intratumoral heterogeneity within tumor microenvironment (TME), providing insights into understanding the mechanisms for tumor progression and developing novel therapeutic strategies for cancers [10]. Using scRNA-seq, several single-cell signatures have been identified as biomarkers of early malignancy, rare tumor types, and widespread reprogramming in TME in GAC [11–13]. However, applying this technology to AEG has been limited due to its rarity compared to GAC. The properties of heterogeneity at the single-cell level remain unknown in AEG. In this study, we aim to investigate AEG and GAC to examine the intra- and inter-tumoural heterogeneity of carcinoma cells and TME, with the goal of improving our understanding of AEG using scRNA-seq.

## Materials and Methods

### Patients and sample collection

Four treatment-naïve patients, who were newly diagnosed with AEG, were enrolled in this study. Prior to their surgeries, written informed consents were obtained from each patient, and fresh samples were collected post-surgery. Approval for this study was granted by the Ethics Committee of Guangdong Provincial People’s Hospital.

### Preparation of single-cell suspensions

Fresh tumor samples were immediately processed upon collection by enzymatic digestion and mechanical dissociation to generate single cell suspensions. Each tumor was cut into small 1-mm3 pieces using D10 resuspension buffer containing culture medium (DMEM medium; Gibco™, USA; Cat. no. 11965092) with 10% fetal bovine serum (FBS; Gibco™; Cat. no. 10099141). Type II (Thermo Fisher, USA; Cat. no. 17101015) and IV (Thermo Fisher; Cat. no. 17104019) enzymes were used for 30 min on a rotator at 37 °C. The digested mixture was then filtered through a 40-μm cell strainer (BD Biosciences, USA; Cat. no. 352340) to obtain dissociated cells. After centrifugation at 400 X g for 5 min, the pelleted cells were resuspended in 0.8% NH4Cl red blood cell lysis buffer and incubated on ice for 10 min. The dissociated cells from the tumor were washed twice with DPBS (Gibco™; Cat. no. 14190250) and resuspended in sorting buffer (1X DPBS supplemented with 0.04% BSA; Sigma-Aldrich, USA; Cat. no. 9048468). Viable cells were collected using fluorescence-activated cell sorting (FACS; BD FACSAria III; BD Biosciences) with negative staining of propidium iodide (PI; Thermo Fisher, Cat. no. P1304MP). At least 300,000 cells were collected for each tissue sample.

### Library construction for single-cell gene expression and T cell receptors (TCR) profiling

Immune repertoire measurement and gene expression at single-cell resolution were conducted using Chromium Single Cell V(D)J Reagent Kit (10x Genomics, USA) following the manufacturer’s instructions. Briefly, the sorted cells were washed twice with the sorting buffer. Cell viability and number were determined using Trypan Blue (Thermo Fisher; Cat. no. 15250061) exclusion assay. Appropriate volume of cell suspension with a concentration of 700-1,200 cells/µl were loaded in each channel, targeting a capture of 8000 cells per sample, which were further mixed with barcoded gel beads on a Chromium Controller (10x Genomics). After reverse transcription reaction, cDNA amplification for 14 cycles was conducted on a thermal cycler (C1000; Bio-Rad, USA). The post-amplification cDNA was used as template to further enrich TCR fragments. Sequencing libraries for cDNA and TCR were separately constructed according to the instructions. The average fragment size of a library was quantitated using Qseq100 (Bioptic; Taiwan).

### Next-generation sequencing and data processing

After generating pair-end reads of 150 bp, each DNA library was loaded into a sequencing lane on a HiSeq X system (Illumina, USA). The raw data in Binary Base Call (BCL) format was then converted to FASTQ files using bcl2fastq (version v2.19.0.316, Illumina). The Cell Ranger pipelines (version 3.0.1; 10x Genomics) were utilized to align sequencing reads in the FASTQ files to reference genomes and generate feature-barcode matrices for single-cell 5’-gene expression data and TCR-enriched data from the same cDNA library. The gene expression data was mapped to the human genome reference sequence (GRCh38; http://cf.10Xgenomics.com/supp/cell-exp/refdata-cellranger-GRCh38-1.2.0.tar.gz), while the TCR enriched data was mapped to the VDJ reference sequence (http://cf.10Xgenomics.com/supp/cell-vdj/refdata-cellranger-vdj-GRCh38-alts-ensembl-2.0.0.tar.gz) for cDNA and TCR sequencing reads, respectively. This process was carried out using the Cell Ranger count and Cell Ranger vdj implemented in the pipelines.

### Single-cell gene expression quantification and determination of cell types

We combined the gene expression matrices for all remaining cells and converted them into a Seurat object using the R package Seurat (version 3.0.1, https://satijalab.org/seurat). For quality control, cells that had either less than 101 UMIs or expression of fewer than 501 genes, or over 15% UMIs linked to mitochondrial genes, were removed from the dataset. From the remaining cells, gene expression matrices were generated with log normalization and linear regression using the NormalizeData and ScaleData function of the Seurat package.

To identify doublets in our data, we utilized the R package “DoubletFinder” (https://github.com/chris-mcginnis-ucsf/DoubletFinder). Essentially, a doublet is defined as a single-cell library representing more than one cell, and by examining known markers, we determined that the offending cluster consisted of doublets of more than one cell type, as no cell type is known to strongly express both markers at the same time. We removed doublets in each sample individually, with default parameters used except for an expected doublet rate of 0.05. The remaining cells were identified as single cells.

To address potential batch effects resulting from independently processed samples and high-dimensional variables in single-cell sequencing data, we employed the Harmony and RunUMAP function implemented in Seurat to reduce dimensionality and remove batch effect. Cell clusters were identified using the FindClusters function in Seurat with a K parameter of 20, and default parameters were used otherwise. We annotated the clusters as different major cell types based on their average gene expression of well-known markers. Specifically, CD4^+^ T cells were identified using PTPRC, CD3D, and CD4; CD8^+^ T cells were identified using PTPRC, CD3D, and CD8A; myeloid cells were identified using CD14 and ITGAX encoding CD11C; malignant cells were identified using EPCAM and KRT family genes; B cells were identified using CD19 and MS4A1; cancer-associated fibroblasts (CAF) were identified using COL1A1, and NK cells were identified using FCGR3A and NCAM1.

In addition to the previous steps, we further identified sub-clusters and annotated them as different specific cell subtypes based on the average expression of respective gene sets in each major cell type. To identify marker genes for each sub-cluster within the major cell types (CD4^+^ T, CD8^+^ T, NK, B, CAF, myeloid, and malignant cells), we compared the expression profiles of the sub-cluster with those of other sub-clusters using the Seurat FindAllMarkers function. Differential expression analysis was performed using the default two-sided non-parametric Wilcoxon rank sum test, comparing all genes in the two datasets. A gene was considered significantly differentially expressed if it had a Bonferroni-adjusted P-value less than 0.05 and an average natural logarithm (ln) fold-change of expression of at least 0.1 and 0.25 for malignant cells and other cells, respectively. We removed clusters that had multiple well-defined marker genes of different cell types and an elevated number of UMIs, as they were considered cell contamination in downstream analysis.

### Pathway enrichment analysis

To compare the difference of signaling pathway enrichment between two clusters, we performed the gene set enrichment analysis (GSEA; version 3.0) using the molecular signatures database v7.0 [14]. To gain functional and mechanistic insights of a cell cluster, we performed gene set variation analysis (GSVA, version 1.34.0), using the molecular signature database v7.0 [14].

### TCR repertoire analysis

The outputs of CellRanger vdj included the assembled nucleotide sequences for both α and β chains, the coding potential of the nucleotide sequences (that is productive or not), the translated amino acid sequence, the CDR3 sequences, and the estimated UMI value of α or β chains. Only cells with UMI values larger than 1 for α and β chains were kept. The dominant TCR of a single cell was defined based on an in-frame TCR α-β pair. If one clonotype defined as a unique in-frame TCR α-β pair was present in at least two cells, this clonotype would be considered clonal, and the number of cells with such dominant α-β pair indicated the degree of clonality of the clonotype.

### inferCNV analysis

To identify malignant cells, we identified evidence for somatic alterations of large-scale chromosomal copy number variants, either gains or losses, in a single cell using inferCNV (https://github.com/broadinstitute/inferCNV), in addition to the expression of EPCAM. The raw single-cell gene expression data were extracted from the Seurat object according to the software recommendation. We preformed inferCNV analysis with the default parameters.

### Developmental trajectory inference

To characterize the potential process of cell functional changes and determine the potential lineage differentiation among diverse cells, we performed trajectory analyses for B and CD8^+^ T cells, using Monocle3 [15] (version 0.0.2; http://cole-trapnell-lab.github.io/monocle3/). Seurat output data for specific clusters was fed directly into Monocle3, followed by removal of batch effect using align_cds function. Cell trajectory was calculated by using learn_graph function. Cell differentiation trajectory was inferred after dimension reduction and cell ordering with the default parameters implemented in Monocle3. Gene expression along pseudotime data was extracted from the output of plot genes in pseudotime function and was used to plot genes along pseudotime of lineages using ggplot2 (version 3.3.3) in R package.

### Cell-cell interaction analysis

To compare the differences of mutual regulatory network among cells from different sources, we used CellChat [16] (version 1.0.0; https://github.com/sqjin/CellChat) with the normalized counts by Seurat and the standard pre-processing functions identifyOverExpressedGenes, identifyOverExpressedInteractions, and projectData. As for the reference database, we included the secreted signalling pathways and the precompiled human protein-protein and extracellular matrix (ECM)-receptor Interactions as a priori network information. The core functions computeCommunProb, computeCommunProbPathway and aggregateNet were applied with standard parameters and fixed randomization seeds. The function netAnalysis computeCentrality was applied on the netP data slot to compute the network centrality scores.

### Statistical analysis

Statistical analyses were performed using R (version 4.1.2), with methods as described in the Figure legends. *P* < 0.05 was considered as statistical significance

## Results

### Landscape profiling of AEG by scRNA-seq

**Fig. 1:**
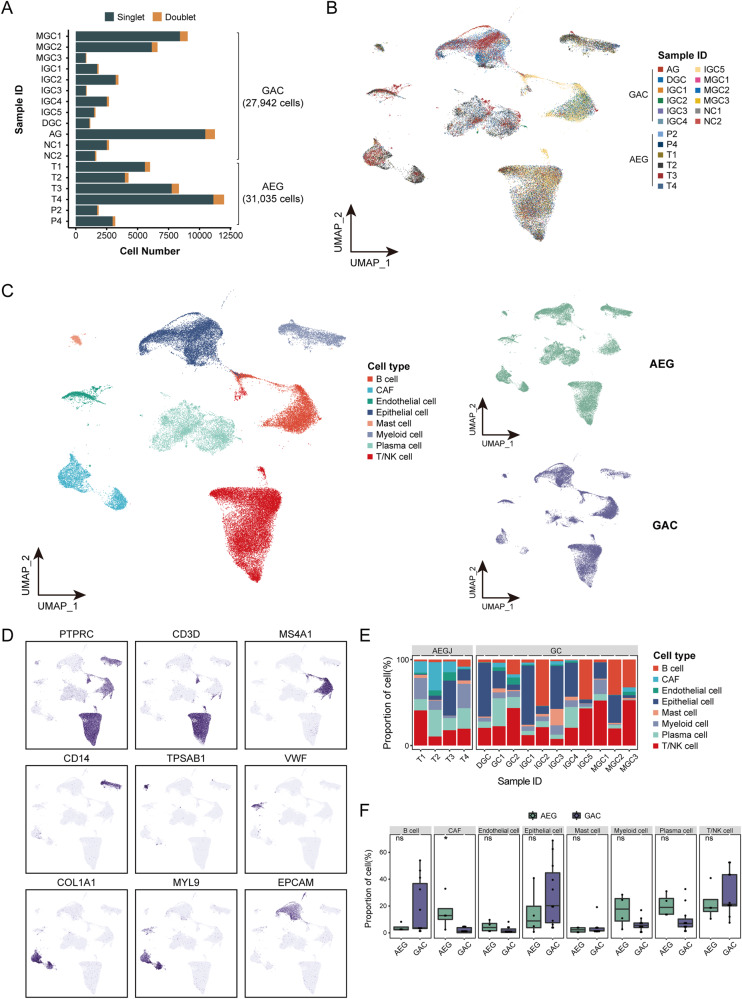
The landscape profiling of single cells in AEG and GAC tumors. **A** The number of cells for each sample, including singlets and doublets. **B** UMAP plot showing cells derived from each AEG and GAC sample. **C** UMAP plot showing cells grouped into eight major cell types (left panel) from either AEG or GAC tumors (right panel). **D** Expression of canonical marker genes to define the major cell types. **E** The proportion of major cell types in each AEG and GAC sample. **F** Comparison of the proportion for each major cell type between AEG and GAC using Wilcoxon rank-sum test. ^ns^*P* ≥ 0.05, ^*^P < 0.05.

In order to gain insight into the heterogeneous cell composition of AEG, we performed single-cell RNA transcriptome sequencing coupled with immune repertoire sequencing (TCRs) on viable cells derived from four treatment-naïve AEG tumor samples and two adjacent normal samples (Supplementary Fig. 1 and Supplementary Table 1). Additionally, we collected a publicly available scRNA-seq dataset from patients with GAC for further integrated analysis [12]. After performing quality control and data integration, we identified a total of 58,977 cells from all samples, which included 31,035 cells from AEG patients and 27,942 cells from GAC patients (Fig. 1A). On average, each cell from AEG patients yielded about 1966 genes and 8670 unique molecular identifiers (UMIs), indicating sufficient coverage and representative of transcripts.

Next, we utilized Seurat to classify cells into groups of cell types with similar expression profiles. Through graph-based clustering, the distribution of cell clusters was consistent across samples (Fig. 1B), suggesting that there were no discernible batch effects associated with inherent variance across individuals during our analyses. We were able to identify eight major cell types based on the expression of canonical markers, including B cells, CAFs, endothelial cells, epithelial cells, mast cells, myeloid cells, plasma cells, and T/NK cells (Fig. 1C, D).

All major cell types were observed with variable cell fractions among samples (Fig. 1E), indicating the common occurrence of infiltrating immune cells and individual heterogeneity of cell proportions in AEG tumors as in GAC. Moreover, we observed different cell compositions between these two types of cancers, with significant increase of CAFs in AEG samples (Fig. 1F), which suggests a distinct cellular architecture of AEG TME compared with GAC.

### Heterogeneous malignant cell clusters in AEG

**Fig. 2:**
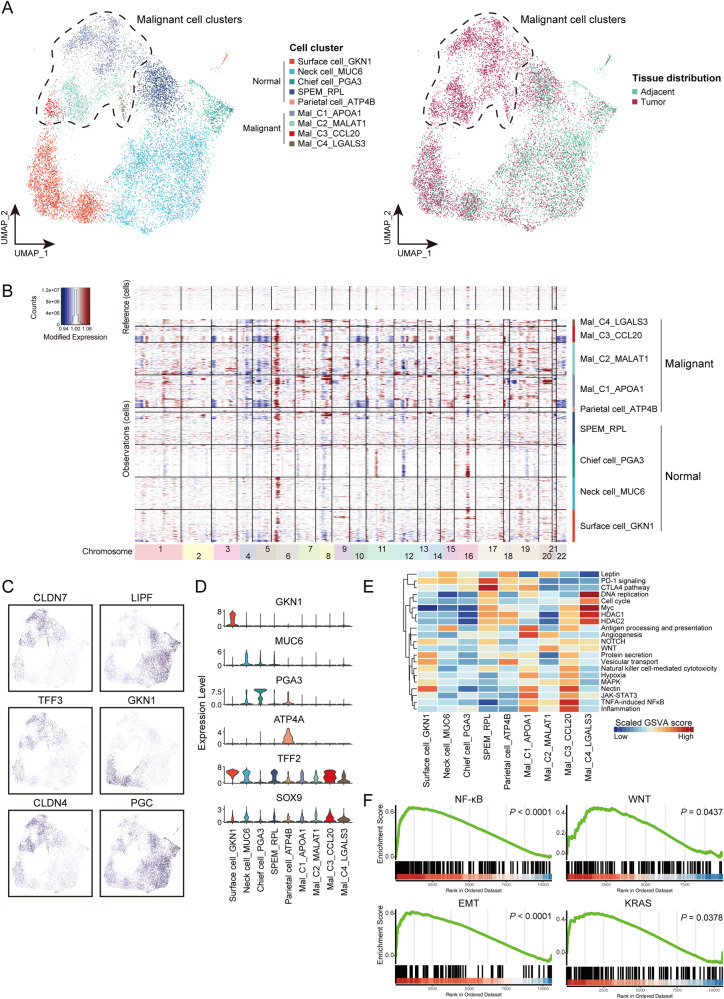
Characterization of the heterogeneous malignant cell clusters in AEG. **A** UMAP plot showing malignant and normal epithelial cell clusters (left panel) derived from either adjacent or tumor tissues (right panel). **B** Heatmap showing the large-scale chromosomal CNVs predicted from inferCNV to identify malignant cell clusters. **C** Expression of marker genes to distinguish malignant and normal epithelial cells. **D** Expression of histological markers of gastrointestinal tract to identify normal epithelial cell clusters. **E** Heatmap showing the distinct enrichment of functional pathways for malignant and normal epithelial clusters. **F** GSEA analysis revealing enrichment of oncogenic signaling pathways in malignant cells compared to normal epithelial cells.

We identified a total of 11,734 epithelial cells, which were clustered into nine subclusters with their expression of distinct gene expression signatures, including five normal epithelial cell clusters and four malignant cell clusters (Fig. 2A). The malignant cell clusters were characterized based on the large-scale chromosomal copy number variations using inferCNV (Fig. 2B), as well as their in-situ location in tumors in comparison to the common distribution of normal cells both in tumor and adjacent samples (Fig. 2A). Supportively, we observed distinct expression of well-known marker genes in these cell clusters as documented previously (*CLDN7*, *TFF3*, and *CLDN4* for malignant cells, and *LIPF*, *GKN1*, and *PGC* for normal epithelial cells; Fig. 2C), indicating the robustness of our annotation. In addition, the cell type of each normal epithelial cluster was identified according to the expression of canonical markers for histological characteristics of gastrointestinal tract (Fig. 2D).

Through functional enrichment analysis via GSVA, malignant cell clusters were observed with predominant activation of various signaling pathways, including antigen presentation and angiogenesis in Mal_C1_APOA1, inflammation signaling in Mal_C3_CCL20, and cell proliferation-associated DNA replication, cell cycle and Myc signaling in Mal_C4_LGALS3 (Fig. 2E). Moreover, GSEA analysis revealed common activation of multiple oncogenic signaling pathways, such as NF-κB, WNT, epithelial-mesenchymal transition (EMT), and KRAS pathways, in malignant cells compared with normal epithelial cells (Fig. 2F). Taken together, these observations suggest the heterogeneity of AEG malignant cells with diverse biological functions and intrinsic malignant nature.

### Immune dysfunction and development of T cells in AEG tumors

**Fig. 3:**
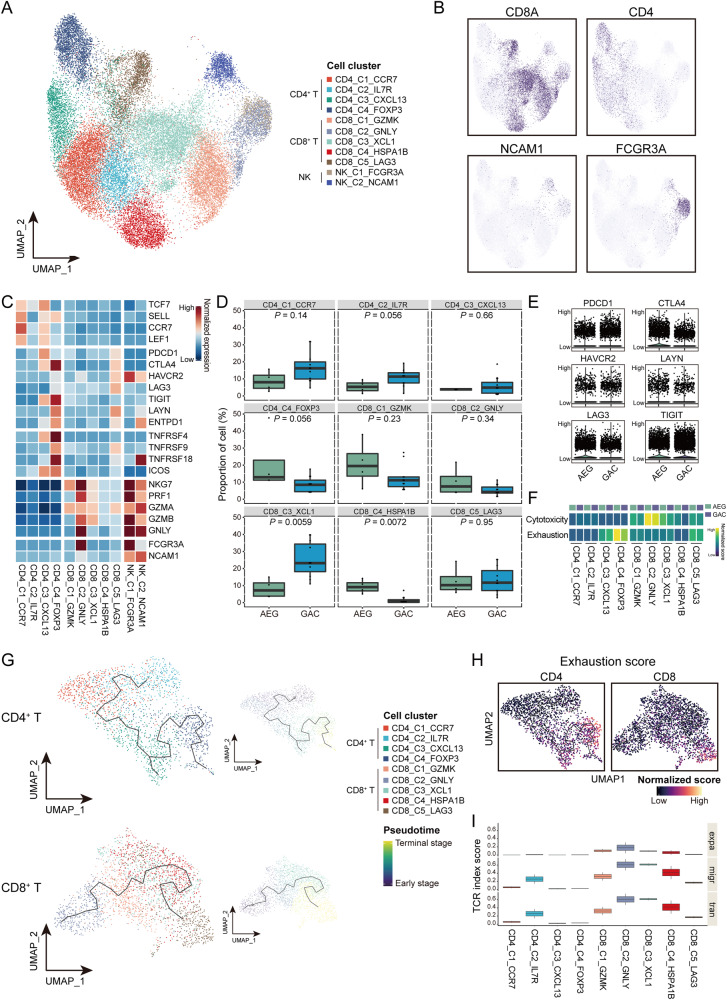
The exhausted state of T cells in the tumor microenvironment of AEG. **A** UMAP plot showing CD4^+^ T, CD8^+^ T, and NK cell clusters. **B** Expression of canonical marker genes for CD4^+^ T, CD8^+^ T, and NK cell. **C** Expression of marker genes to identify naïve, exhausted, regulatory, and effector T cells, as well as NK cells. **D** Comparison of the proportion for each CD4^+^ and CD8^+^ T cell cluster between AEG and GAC using Wilcoxon rank-sum test. **E** Expression of exhausted molecules in T cells derived from either AEG or GAC tumors. **F** Calculated cytotoxicity and exhaustion scores for each T cell cluster derived from either AEG or GAC tumors. **G** Pseudotime trajectories of the developmental paths for CD4^+^ (top panel) and CD8^+^ T cells (bottom panel) in AEG. **H** Increasing exhaustion scores along the developmental trajectories of CD4^+^ and CD8^+^ T cells. **I** The TCR index scores of expansion (expa), migration (migr), and transition (tran) for each CD4^+^ and CD8^+^ T cell cluster.

A total of 19,548 T/NK cells were divided into 11 cell clusters, including four CD4^+^ T, five CD8^+^ T, and two NK cell clusters (Fig. 3A-C). We observed distinct cell fractions of T cell clusters between AEG and GAC tumors, with a significantly lower and higher proportion for CD8_C3_XCL1 and CD8_C4_HSPA1B T cells, respectively (Fig. 3D). Higher expression levels of exhausted molecules, including *PDCD1*, *CTLA4*, *HAVCR2*, *LAYN*, *LAG3*, and *TIGIT*, in the T cells in AEG in comparison with those in GAC (Fig. 3E). We calculated exhaustion scores for CD8^+^ and CD4^+^ T clusters based on the expression of a set of immunosuppressive molecules, which revealed the highest exhaustion in regulatory T cells (CD4_C4_FOXP3) and exhausted T cells (CD8_C5_LAG3), with further increase in AEG (Fig. 3F).

We performed pseudotime trajectory analyses using Monocle3 to investigate the developmental links for CD4^+^ and CD8^+^ T cells in AEG. Indeed, we observed a development branch from naïve/central memory (CD4_C1_CCR7 and CD4_C2_IL7R) to regulatory T cells (CD4_C4_FOXP3) for CD4^+^ T cells, and from cytotoxic (CD8_C2_GNLY) to exhausted T cells (CD8_C5_LAG3) for CD8^+^ T cells (Fig. 3G and Supplementary Fig. 2A) in both AEG and GAC, along which we observed increasing exhausted scores for T cells at terminal stages (Fig. 3H and Supplementary Fig. 2B). Combining with TCR information for these T cells, we observed large scales of TCR clones for CD4^+^ and CD8^+^ T cells at early stages (CD4_C2_IL7R and CD8_C2_GNLY), in comparison to the minimal TCR clones in those at terminal stages (CD4_C4_FOXP3 and CD8_C5_LAG3) (Fig. 3I). These observations suggest the potential developmental process for T cells in AEG, with enhanced exhaustion and reduced cloning along the pseudotime paths.

### High infiltration of tumorigenic CAFs in AEG TME

**Fig. 4:**
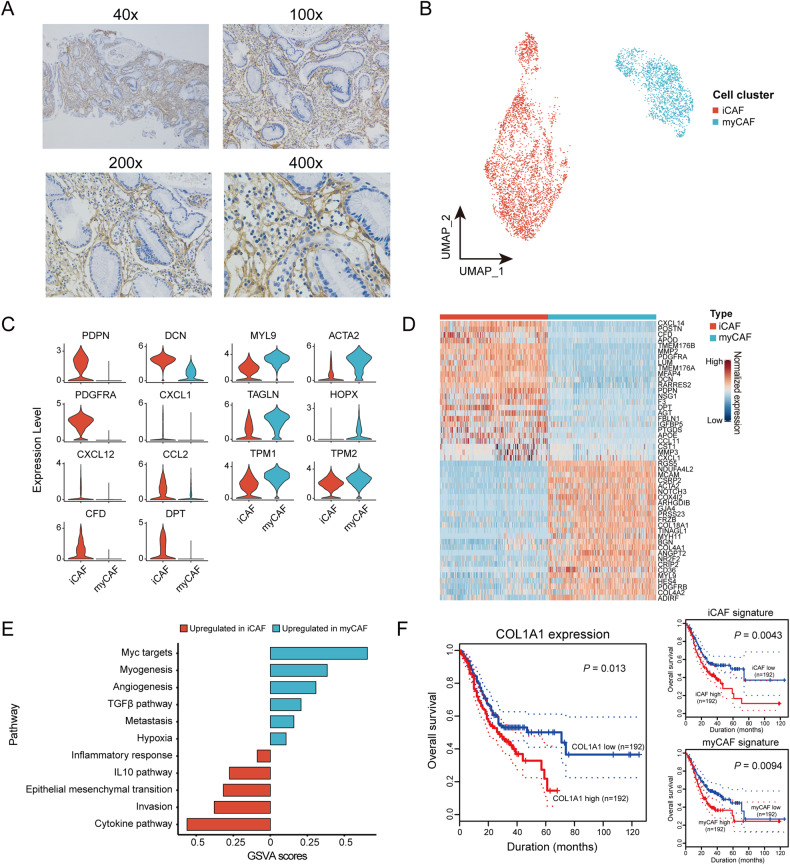
Infiltration of tumorigenic CAFs in the tumor microenvironment of AEG. **A** Representative images of immunohistochemistry staining showing the protein expression of COL1A1 in AEG tumors. **B** UMAP plot for two cell clusters of CAFs. **C** Expression of marker genes to identify iCAF and myCAF. **D** Heatmap showing the differential expressed genes between iCAF and myCAF. **E** Distinct enrichment of signaling pathways between iCAF and myCAF. **F** Survival curves of patients stratified according to the expression of *COL1A1* or signatures of two CAF subclusters. Survival was measured using the Kaplan-Meier method and *P* values were calculated using log-rank test.

A huge proportion of CAFs were identified in AEG tumors according to their marker genes, *COL1A1* (Fig. 1D and Supplementary Fig. 3A, B), which was significantly higher than that in GAC (Fig. 1F). Supportively, immunohistochemistry staining assay of COL1A1 protein expression corroborates the high infiltration of CAFs in AEG tumors (Fig. 4A). Subsequently, these CAFs were grouped into two subclusters, inflammatory subgroup (iCAF) and myofibroblastic subgroup (myCAF) (Fig. 4B), with their high expression of marker genes and distinct transcriptional signatures (Fig. 4C, D). GSVA analysis revealed the predominant enrichment of inflammatory response, IL10 pathway, EMT, tumor invasion, and cytokine pathway in iCAFs, and Myc, myogenesis, angiogenesis, TGFβ signaling, metastasis, and hypoxia in myCAFs (Fig. 4E), suggesting their critical roles on tumor development for AEG in different manners. In addition, pseudotime trajectory analyses revealed the developmental trend from myCAF to iCAF in both AEG and GAC (Supplementary Fig. 3C), suggesting a similar manner for CAF development. Notably, using an independent sample cohort from TCGA dataset with bulk transcriptome data and corresponding prognostic information, high expression of CAF marker (*COL1A1*) was associated with poor overall survival (OS; Fig. 4F). Moreover, survival analyses also revealed that patients with higher levels of signature scores for both iCAFs and myCAFs had worse survival (Fig. 4F). These observations suggest high infiltration of CAFs, including iCAFs and myCAFs, to be an unfavorable prognostic marker in AEG.

### A central regulatory role of CAFs in the intercellular communication network of AEG TME

**Fig. 5:**
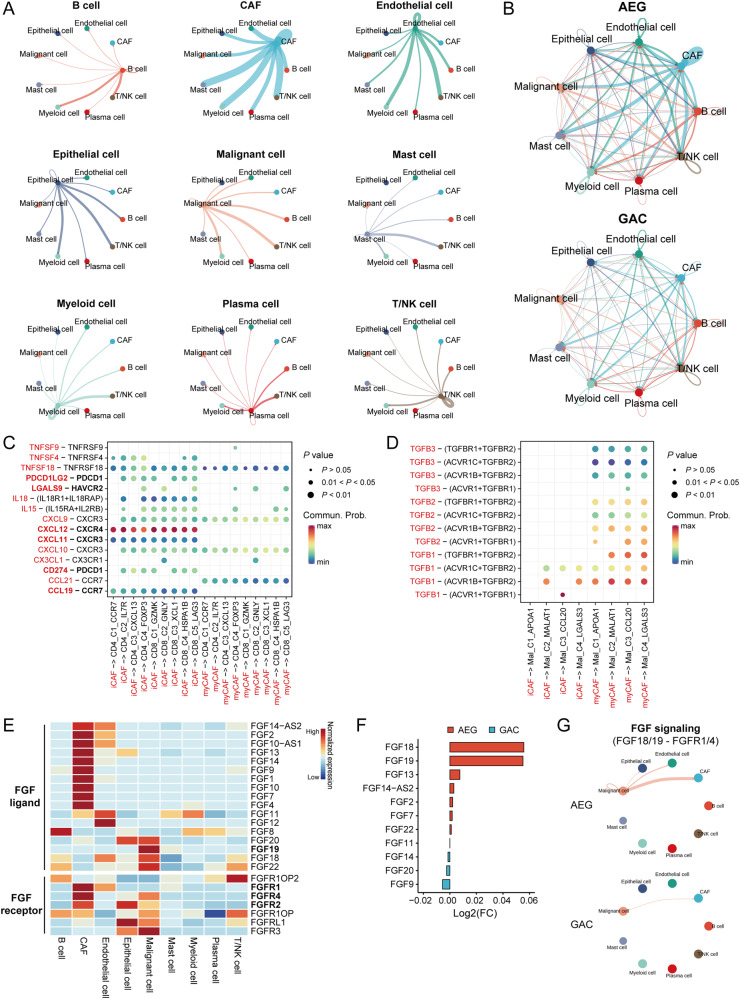
The cellular interaction network of AEG TME. **A** Connection graphs showing each major cell type as a source to interact with the other cell types. **B** Connection graphs showing the cellular interactions among major cell types within the TME of either AEG or GAC. **C** Dot plot showing the co-inhibitory, co-stimulatory, and chemokine interactions between CAFs and T cells. **D** Dot plot showing the interactions of TGFβ signaling between CAFs to malignant cells. **E** Expression of FGF ligands and receptors among major cell types. **F** The differential expression of FGF ligands in malignant cells between AEG and GAC tumors. **G** Connection graphs showing the intensity of FGF-FGFR interactions in AEG and GAC tumors.

To explore the cellular communication network in AEG, we evaluated potential ligand-receptor binding pairs between any two cells using CellChat. Among broad intercellular interactions, we observed the most intensive interactions between CAFs and the other cell types (Fig. 5A). Notably, stronger cellular interactions associated with CAFs were observed in AEG, in comparison to the weak interactions in GAC (Fig. 5B), suggesting a central regulatory role of CAFs in the cellular network of AEG TME. Specially, iCAFs interacted with tumor-infiltrating T cells through CXCL9/10/11-CXCR3, CXCL12-CXCR4, and CCL19-CCR7, suggest that these iCAFs might recruit T cells and shape the immune landscape of AEG TME through multiple chemotactic regulation (Fig. 5C). We also observed intensive interactions of CD274-PDCD1, PDCD1LG2-PDCD1, and LGALS9-HAVCR2 between iCAFs and tumor-infiltrating T cells (Fig. 5C), which are well-characterized immune checkpoint axes, suggesting their immunosuppressive capability on T cells. Notably, myCAFs had high expression of *TGFB2* and *TGFB3* showing significant interactions with four malignant cell clusters, which are key molecules in the oncogenic TGFβ signaling pathway (Fig. 5D), suggesting a potential contribution of myCAFs on the development of malignant cells.

### Stimulation of CAFs development via FGF-FGFR axis

Given that fibroblast growth factors (FGFs) and their receptors (FGFRs) being the major growth factor and receptor axis controlling the survival and differentiation of fibroblasts [17], we examined and observed unique expression of multiple FGF family ligands (*FGF18*, *FGF19*, *FGF20*, and *FGF22*) in malignant cells, as well as the high expression of their corresponding receptors (*FGFR1*, *FGFR2*, and *FGFR4*) in CAFs (Fig. 5E). Interestingly, malignant cells from AEG had significantly higher expression of several FGF family ligands, such as *FGF18* and *FGF19*, compared to the ones from GAC (Fig. 5F). Moreover, CellChat analysis revealed the stronger cellular interactions of FGF18/19-FGFR1/4 between malignant cells and CAFs in AEG (Fig. 5G). These observations together suggest a contribution of malignant cells to the stimulation of CAF development in AEG via FGF-FGFR axes, thereby leading to the high infiltration of CAFs in AEG tumors.

## Discussion

Here for the first time to our knowledge, we performed transcriptome analysis at the single-cell level for the TME of AGE and delineated a cellular landscape of 31,035 cells within the complex interaction network. Through our analysis, we identified four malignant epithelial cell clusters with distinct transcriptional signatures and tumorigenic functions. Besides, we observed the enhanced exhaustion and reduced cloning of the tumor-infiltrating T cells along their developmental process. Notably, we detected high infiltration of CAFs in AEG TME, which might serve as a central regulatory role in the cellular interaction network and thereby induce the development of malignant cells as well as exhaustion of T cells. Moreover, our analysis revealed malignant cells with high expression of FGF molecules and intensive interactions of FGF-FGFR axis with CAFs, potentially contributing to the accumulation of tumorigenic CAFs in AEG tumors.

To date, the tumor heterogeneity and immune landscapes of gastrointestinal tumors have been reported through multiple large-scales scRNA-seq studies [18–22]. For instance, Zhang and colleagues constructed a single-cell atlas from gastric antral mucosa biopsy samples of patients spanning a cascade of gastric premalignant lesions and early gastric cancer, providing insights into the pathogenic mechanism of GAC [18]. Li and colleagues focused on the heterogeneity of the GAC TME, revealing the significant variability in abundance and expression signatures among tumor epithelial cells and other TME cell subsets, especially the diversity of CAFs, which regulate different biological functions within the TME [19]. Sun et al. present a comprehensive single-cell transcriptome atlas of GAC, described a detailed and complex taxonomy of immune, stromal, and epithelial subsets, and indicate that the stromal cells in the tumor tissue undergo a significant transformation and exhibit extensive tumor-promoting features [20]. Li et al. analyzed the microenvironment of esophageal squamous cell carcinoma (ESCC) using single-cell transcriptome sequencing and revealed prominent heterogeneity in most of the cell types in ESCC stoma, particularly immune cells (myeloid and T cells) and fibroblasts [21]. Cheng et al. performed comprehensive genomic, transcriptomic, proteomic, and phosphoproteomic analyses of tumor tissues derived from 103 AEG patients, which contribute to patient stratification at molecular aspect and provide valuable resources for understanding tumorigenic mechanisms and developing precision treatment strategies for AEG [22]. However, the immune landscape of AEG TME underlying mechanisms of the extent of cellular heterogeneity, the dynamics of distinct biological states, and their functional impact on the tumor ecosystem remain largely uncharacterized.

Tumor immune microenvironment plays a crucial role in determining the effectiveness of PD-1 immunotherapy, with the expression level of PD-L1 in tumor cells and the density and phenotype of tumor-infiltrating T lymphocytes being important factors [23, 24]. In particular, the response rate to PD-1 immunotherapy is higher in PD-L1 positive cases compared to PD-L1 negative cases [25]. Moreover, an increased proportion of exhausted T lymphocytes in tumor-infiltrating T lymphocytes also negatively impacts the efficacy of PD-1 immunotherapy [26]. Currently, PD-1 immunotherapy combined with chemotherapy is widely used as a first-line treatment option for esophageal adenocarcinoma, AEG, and GAC in many countries. However, recent clinical trial data shows that AEG patients with a combined positive score of PD-L1 greater than or equal to 5 points do not benefit from PD-1 immunotherapy [8]. Although the mechanisms for the failure of PD-1 therapy in AEG are not yet fully understood, this study aimed to compare and analyze the TME of AEG and GAC. Single-cell sequencing analysis of AEG and GAC was performed, and network gastric cancer single-cell sequencing data were integrated. The results showed that the proportion of exhausted T lymphocytes was significantly higher in AEG than in GAC, leading to dysfunction of T lymphocytes and inability to effectively kill tumor cells. Additionally, the proportion of CAFs was also higher in AEG, which contributes to solid tumors’ resistance to immunotherapy through various ways [27]. Overall, these findings highlight the importance of understanding the TME in predicting the effectiveness of PD-1 immunotherapy, particularly in AEG patients who may not benefit from the treatment.

Immunotherapy has emerged as a promising treatment option for gastrointestinal cancer [28], with its therapeutic efficacy significantly linked to the tumor immune microenvironment [29]. On one hand, the response of immunotherapy heavily relies on the influence exerted by the tumor immune microenvironment. On the other hand, studies have shown that immunotherapy can also modify the tumor immune microenvironment [30]. Single-cell sequencing technology holds great potential in characterizing the AEG TME and enhancing its responsiveness to immunotherapy. Additionally, comparing the pre- and post-immunotherapy characteristics of the AEG TME may unlock the underlying mechanisms of immunotherapy’s antitumor activity. Moreover, using single-cell sequencing to compare AEG cases with distinct treatment responses can help unravel the interplay between immunotherapy and tumor immune components, paving the way for personalized treatment of AEG patients.

AEG has long been considered a distinct subtype of gastric cancer that shares similar treatment strategies. However, previous data have demonstrated significant differences in therapeutic response between AEG and GAC [8]. Our research utilized single-cell sequencing technology to elucidate the disparities in TME between AEG and GAC, providing novel scientific evidence for personalized treatment of AEG patients. Additionally, through further single-cell sequencing analysis, we hope to identify the immune components that affect immunotherapy response in AEG and explore the underlying reasons for the poor response of AEG to immune therapy. By uncovering the interplay between AEG TME and immune therapy, our study may provide new therapeutic strategies to enhance the immunotherapeutic response of AEG patients.

### Supplementary information

Supplementary Figures 1-3

Supplementary Table 1

Reproducibility checklist

## Data Availability

The raw sequence data reported in this paper have been deposited in the Genome Sequence Archive for human (GSA-Human) under accession number HRA006006. Code used for all processing and analysis is available upon request.
