# Supplementary material for: Comparative single-cell analysis reveals heterogeneous immune landscapes in adenocarcinoma of the esophagogastric junction and gastric adenocarcinoma
Source: Cell Death Dis. 2024 Jan 5;15(1):15. doi: 10.1038/s41419-023-06388-6 (PMC10770337; doi:10.1038/s41419-023-06388-6)
Supplement: Supplementary file 1 — Supplementary Figures 1-3 [file 41419_2023_6388_MOESM1_ESM.docx]

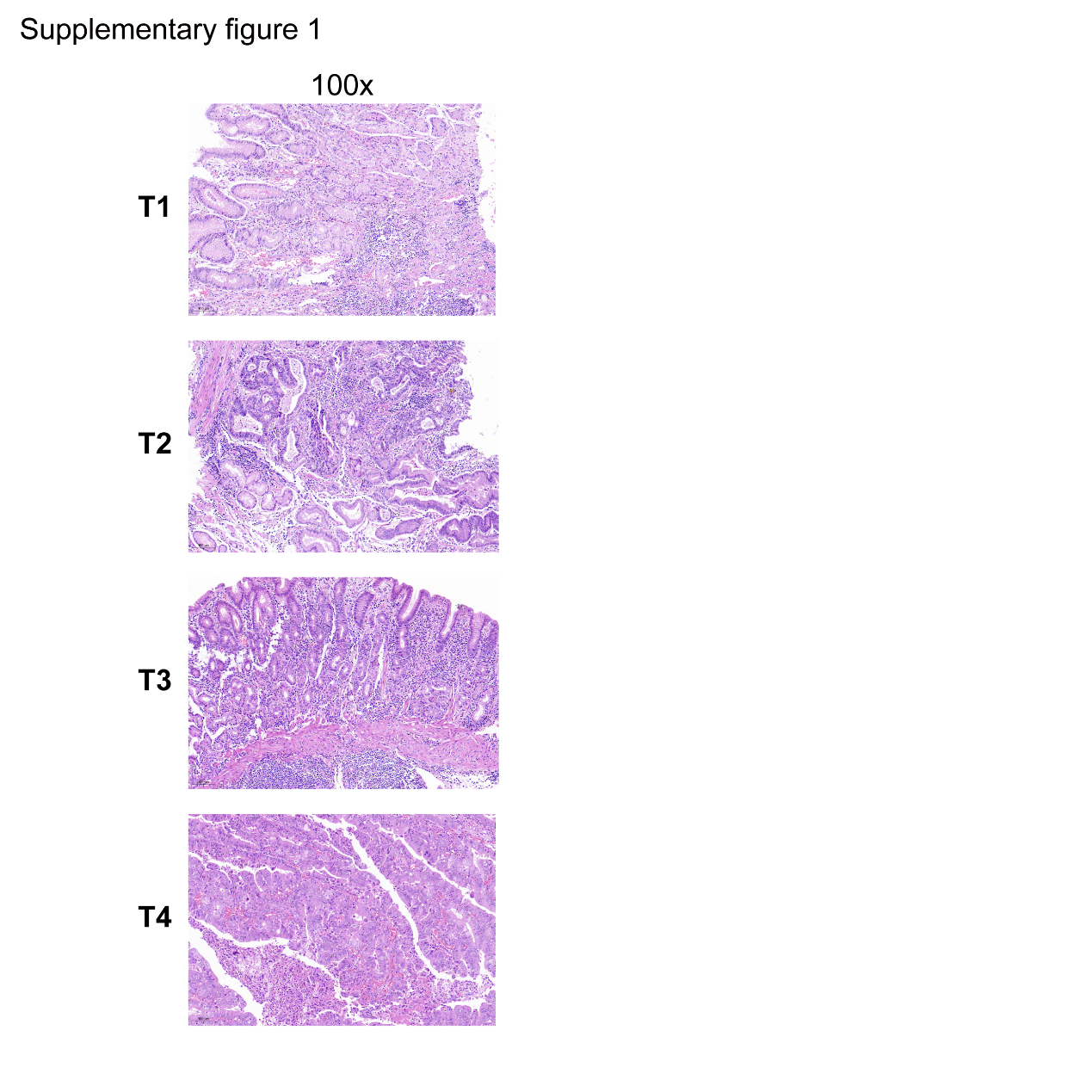


**Supplementary Figure 1. Representative images of HE staining for four AEG samples.**


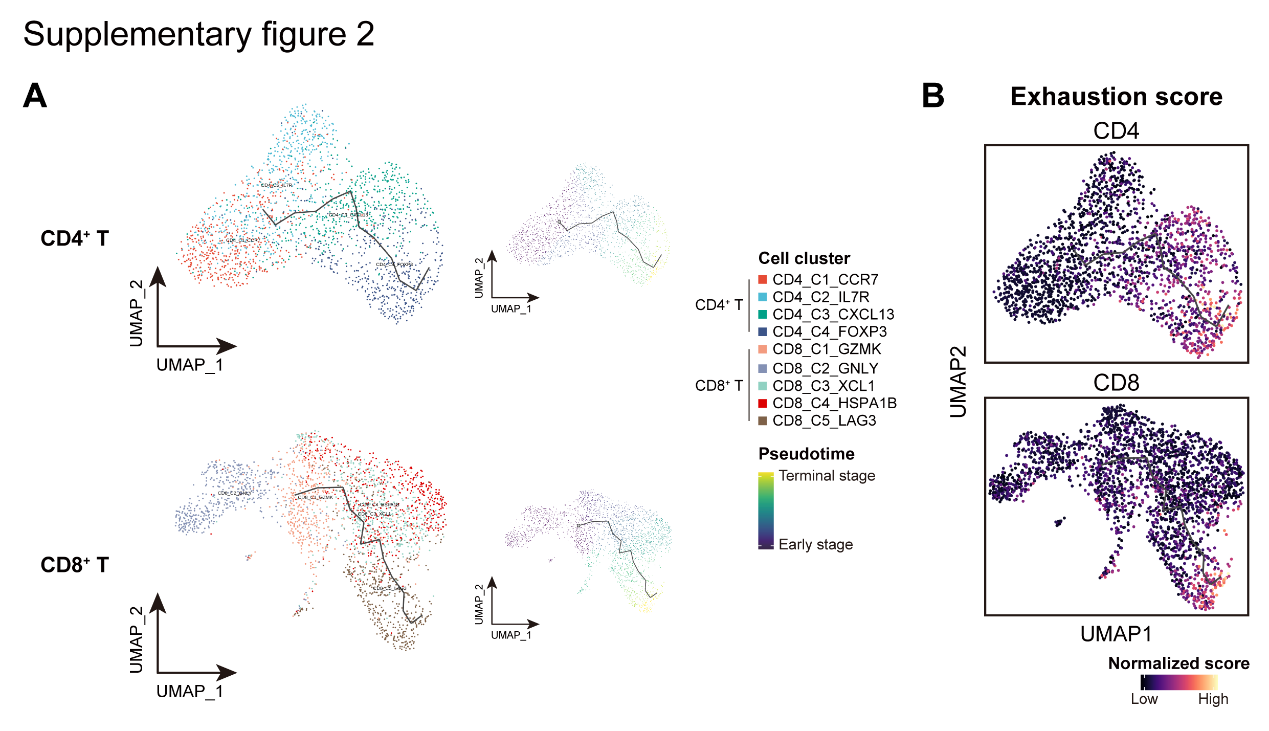


**Supplementary Figure 2. Pseudotime development of T cells in GAC.** **A**. Pseudotime trajectories of the developmental paths for CD4^+^ (top panel) and CD8^+^ T cells (bottom panel) in GAC. **B**. Increasing exhaustion scores along the developmental trajectories of CD4^+^ and CD8^+^ T cells.


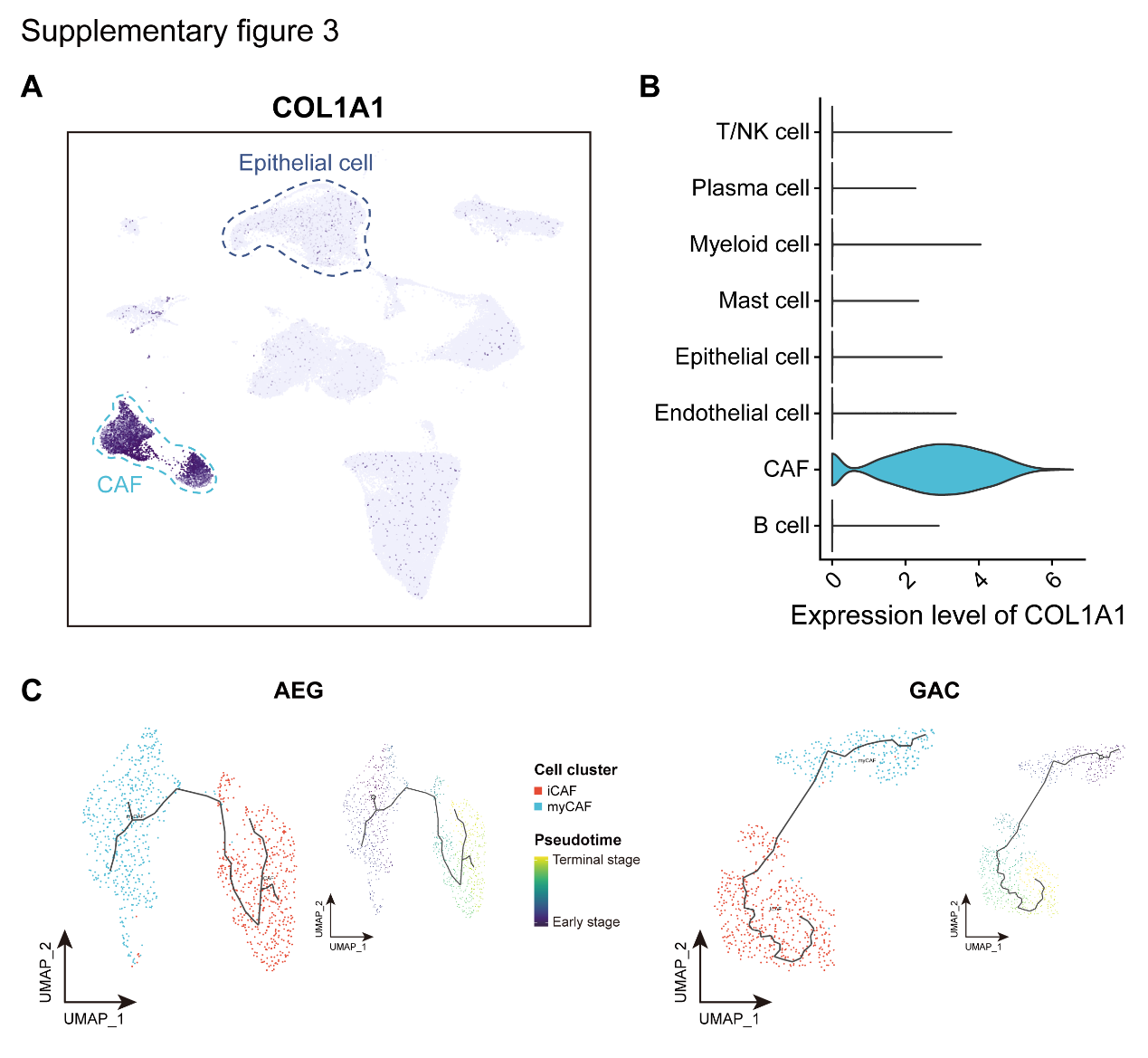


**Supplementary Figure 3. The COL1A1 expression and developmental trend for CAFs.** **A-B**. UMAP plot (**A**) and violin plot (**B**) showing the expression of *COL1A1* in CAFs and other major cell types. **C**. Pseudotime trajectories of the developmental paths from myCAF to iCAF in AEG (left panel) and GAC (right panel).
